# Leveraging AI and Machine Learning to Develop and Evaluate a Contextualized User-Friendly Cough Audio Classifier for Detecting Respiratory Diseases: Protocol for a Diagnostic Study in Rural Tanzania

**DOI:** 10.2196/54388

**Published:** 2024-04-23

**Authors:** Kahabi Ganka Isangula, Rogers John Haule

**Affiliations:** 1 School of Nursing and Midwifery Aga Khan University Dar Es Salaam United Republic of Tanzania

**Keywords:** artificial intelligence, machine learning, respiratory diseases, cough classifiers, Tanzania, Africa, mobile phone, user-friendly, cough, detecting respiratory disease, diagnostic study, tuberculosis, asthma, chronic obstructive pulmonary disease, treatment, management, noninvasive, rural, cross-sectional research, analysis, cough sound

## Abstract

**Background:**

Respiratory diseases, including active tuberculosis (TB), asthma, and chronic obstructive pulmonary disease (COPD), constitute substantial global health challenges, necessitating timely and accurate diagnosis for effective treatment and management.

**Objective:**

This research seeks to develop and evaluate a noninvasive user-friendly artificial intelligence (AI)–powered cough audio classifier for detecting these respiratory conditions in rural Tanzania.

**Methods:**

This is a nonexperimental cross-sectional research with the primary objective of collection and analysis of cough sounds from patients with active TB, asthma, and COPD in outpatient clinics to generate and evaluate a noninvasive cough audio classifier. Specialized cough sound recording devices, designed to be nonintrusive and user-friendly, will facilitate the collection of diverse cough sound samples from patients attending outpatient clinics in 20 health care facilities in the Shinyanga region. The collected cough sound data will undergo rigorous analysis, using advanced AI signal processing and machine learning techniques. By comparing acoustic features and patterns associated with TB, asthma, and COPD, a robust algorithm capable of automated disease discrimination will be generated facilitating the development of a smartphone-based cough sound classifier. The classifier will be evaluated against the calculated reference standards including clinical assessments, sputum smear, GeneXpert, chest x-ray, culture and sensitivity, spirometry and peak expiratory flow, and sensitivity and predictive values.

**Results:**

This research represents a vital step toward enhancing the diagnostic capabilities available in outpatient clinics, with the potential to revolutionize the field of respiratory disease diagnosis. Findings from the 4 phases of the study will be presented as descriptions supported by relevant images, tables, and figures. The anticipated outcome of this research is the creation of a reliable, noninvasive diagnostic cough classifier that empowers health care professionals and patients themselves to identify and differentiate these respiratory diseases based on cough sound patterns.

**Conclusions:**

Cough sound classifiers use advanced technology for early detection and management of respiratory conditions, offering a less invasive and more efficient alternative to traditional diagnostics. This technology promises to ease public health burdens, improve patient outcomes, and enhance health care access in under-resourced areas, potentially transforming respiratory disease management globally.

**International Registered Report Identifier (IRRID):**

PRR1-10.2196/54388

## Introduction

Respiratory diseases impose a significant burden on individuals, communities, and health care systems worldwide. These conditions, which encompass a wide range of disorders such as tuberculosis (TB), asthma, chronic obstructive pulmonary disease (COPD), and lung cancer, not only diminish the quality of life for affected individuals but also lead to substantial economic and health care costs particularly in low- and middle-income countries [1]. Patients with respiratory diseases often struggle with symptoms like coughing, shortness of breath, and chest pain, which can limit their ability to perform daily activities and enjoy a normal life [1,2]. The burden of respiratory diseases extends beyond the physical aspect, as they are frequently associated with emotional distress and psychological challenges, contributing to diminished overall well-being for patients and their families.

From a public health perspective, respiratory diseases account for a significant portion of global morbidity and mortality. The World Health Organization (WHO) reports that respiratory infections alone are responsible for millions of deaths each year, particularly affecting vulnerable populations such as children, the elderly, and individuals with compromised immune systems [3,4]. Moreover, the economic costs associated with respiratory diseases are staggering, encompassing medical expenses lost productivity due to illness, and the burden on health care systems. For instance, the total spending across all respiratory conditions increased by US $71.7 billion in the United States by 2016 from 170.8 billion in 1996 with asthma and COPD contributing US $35.5 billion and US $34.3 billion, respectively, of the total increase [3]. TB, asthma, and COPD are said to be common and rising public health burdens in Africa [5]. Preventive measures such as smoking cessation, improved air quality and vaccination are critical to alleviating this burden, as they can reduce the incidence and severity of respiratory diseases and ultimately improve the overall health and well-being of populations around the world [3]. However, respiratory diseases are relatively neglected in sub-Saharan Africa with no tangible public health programs to address them [5,6]. Consequently, the provision of health care for respiratory diseases appears suboptimal and this was further compromised by the COVID-19 pandemic [5].

Taking TB as an example, the WHO continues to emphasize the need for early detection as a strategic approach for reducing the risk of transmitting the disease to others, improving health outcomes, and reducing distress and economic hardship at the family level [7,8]. Much emphasis is now placed on user-friendly, inexpensive, and contextually feasible diagnostic tools that can effectively identify people who probably have active TB [7,8]. Consequently, countries like Tanzania have started incorporating some of the WHO recommendations about using new user-friendly digital techniques for managing TB into their policies. However, due to the need for improved quality of health care workers with essential knowledge and skills, real adoption of such technologies is still less than intended.

The emergence of the COVID-19 pandemic profoundly impacted the diagnosis and management of other respiratory diseases. With health care systems overwhelmed by the surge in COVID-19 cases, resources, personnel, and laboratory capacities were often diverted away from routine health care services, including the diagnosis of respiratory conditions such as TB, asthma, and COPD [5]. For example, most guidelines recommended limiting the use of pulmonary function tests for fear of transmission of COVID-19 which constrained the use of spirometry testing further compromising the diagnosis of chronic respiratory diseases [9]. Furthermore, the fear of contracting COVID-19 in health care settings led many individuals to postpone or avoid seeking medical care for respiratory symptoms, resulting in delayed diagnoses and exacerbation of preexisting conditions [5]. Diagnostic tests and imaging equipment were sometimes repurposed for COVID-19 testing, leaving fewer resources available for the timely detection of other respiratory ailments. As a result, there has been a concerning backlog in the diagnosis of respiratory diseases, potentially leading to worsened health outcomes and increased burden on health care systems as they work to catch up on missed diagnoses and treatments.

One promising avenue for improving the diagnosis of respiratory diseases is the analysis of cough sounds, which can carry valuable information about the underlying respiratory condition. Most importantly, the recent developments in artificial intelligence (AI) and machine learning (ML) have heightened the need for the application of these powerful tools in the diagnosis of respiratory diseases, particularly, through the development of cough sound classifiers [10,11]. These sophisticated algorithms have the capacity to analyze and interpret patterns within cough sounds, providing valuable insights into a patient’s respiratory health [10-17]. By training AI models on large data sets of cough audio recordings from both healthy individuals and those with respiratory conditions such as TB, asthma, or COPD, it becomes possible to identify distinctive acoustic signatures associated with different diseases [12-17]. These AI-driven cough classifiers can discern subtle variations in cough frequency, duration, intensity, and spectral characteristics, enabling early and accurate disease detection [12-17]. A good example of how AI and ML have been proven useful is the documentation of their effectiveness in Western countries toward the detection of TB [12], COVID-19 [13,14], asthma, COPD [15,16], and even pertussis [17] and their values have extended beyond sound classification to interpreting and analysis of medical imaging for TB detection [18,19]. Moreover, the noninvasive nature of cough audio analysis makes it a convenient and cost-effective diagnostic tool, particularly in resource-constrained settings [12-17]. Patients can record their cough sounds using smartphones or dedicated devices, and these recordings can then be processed by AI algorithms for rapid assessment [20-22]. The potential for remote monitoring is especially valuable in the context of respiratory diseases, as it allows for continuous tracking of a patient’s condition and response to treatment.

Coughing is a common indication of respiratory illnesses, resulting from a forceful release of air from the air passages. Nevertheless, the impact of coughing on the respiratory system is recognized to be variable [23,24]. For instance, lung ailments can lead to either constriction or blockage of the airways, which can affect the sound characteristics of coughing [24]. Furthermore, it has been suggested that the glottis behaves differently under various pathological conditions, allowing for the differentiation of coughs associated with asthma, bronchitis, and pertussis [25]. As a result, the automatic classification of acoustic signals linked to cough to detect respiratory diseases like TB, asthma, and COPD seems to be a reasonable scholarly endeavor to pursue. Research suggests that conditions such as TB, asthma, and COPD exhibit specific temporal patterns and frequencies in coughing, which can be effectively analyzed with advanced technology [26,27].

As AI and ML continue to advance, the accuracy and precision of cough audio classifiers are expected to improve and may outperform standard tests enhancing their use in both clinical practice and public health initiatives. For example, a recent study in Europe indicated that the sensitivity and positive predictive value of the AI-based algorithm were superior to pulmonologist-based diagnostic category allocation in each of the 8 disease groups evaluated [28]. In essence, these technologies hold great promise in revolutionizing the way we diagnose and manage respiratory diseases, offering early intervention, personalized treatment, and improved patient outcomes. However, a major shortfall with the application of AI and ML is that they have not been fully exploited in cough audio classification in rural African contexts. Given the burgeoning interest in leveraging AI or ML for health care advancements, there is a critical need for empirical research to explore their potential applications in rural African settings. The gap in noninvasive, efficient diagnostic tools for respiratory diseases in low-income Africa necessitates urgent attention. This study aims to develop and assess an AI-powered cough audio classifier designed to detect respiratory conditions in rural Tanzania. Through a systematic approach to collecting and analyzing cough sounds in outpatient settings, this research aims to improve disease identification accuracy and efficiency, offering accessible, user-friendly diagnostic solutions to support health care providers in low-income areas.

## Methods

### Design

This is nonexperimental cross-sectional research with the primary objective of collection and analysis of cough sounds from patients with active TB, asthma, and COPD in outpatient clinics to generate and evaluate a noninvasive cough audio classifier. The study combines innovative applications of AI or ML to develop cough audio classifiers using data collected from patients with active diseases and evaluation of the classifiers in routine care against the reference standards. To achieve this, a four-phase research process will be implemented involving (1) cough sound collection for 12 months (phase 1), (2) cough sound analysis and algorithm development for 12 months (phase 2), (3) development of cough audio classifier for 6 months (phase 3), and (4) evaluation of cough audio classifier for 18 months (phase 4). The expected primary outcomes are contextualized user-friendly AI-powered cough audio classifier and a rate of patients with active respiratory diseases notified and linked to treatment. The expected secondary outcomes are health care workers’ experience with a cough audio classifier, including perspectives on the acceptability, feasibility, and cost of using a cough audio classifier. The comparison of the cost incurred to implement the audio classifier will be compared to the standards of care. The feasibility indicators will include the perceived acceptability and satisfaction of health care providers and patients toward the cough audio classifier. Findings from this study will be useful in further refinement of the cough audio classifier and definitive trials in the broader community. The research will increase the pool of health experts with skills in the application of AI or ML developing diagnostic tests in Tanzania. It is expected that the cough audio classifier if found effective during future definitive trials, can be trademarked.

### Settings

The study will take place across the Shinyanga Region of Tanzania. The Shinyanga Region, predominantly rural with less than 5% urban residency, is detailed further in my prior research [29,30]. More specifically, the study will be conducted in 30 facilities including Shinyanga Referral Hospital, Shinyanga Municipal Hospital, Shinyanga District Council Hospital, Kolandoto Hospital, Kahama Municipal Hospital, Jakaya Kikwete Hospital, Kishapu health centre (HC) Mwadui Hospital, Nindo Dispensary, Tinde HC, Samuye HC, Ngokoro HC, Kambarage HC, Imani Dispensary, Inspire Dispensary, Mwawaza Dispensary, Kizumbi Dispensary, Bubiki Dispensary, Polisi Dispensary, Lubaga Dispensary, Consolata Dispensary, Majengo Dispensary, Maganzo Dispensary, Mwamalili Dispensary, Mwamalili Dispensary, Golden Grace Dispensary, Masekelo Dispensary, Kanisa la Kiinjiri la Kilutheri Tanzania Lutheran Dispensary, Old Shinyanga Dispensary, and Chamaguha Dispensary. These sites are purposefully selected considering ownership (public, private, and faith-based organization), location (urban, peri-urban, and rural), level (hospital, health center, and dispensaries), and access to a wealthy number of clients presenting with coughs. The focus on rural settings stems from the commitment of the principal investigator (PI) to enhancing primary health care [29]; challenges in diagnosing TB, asthma, and COPD due to limited diagnostics; the value of co-designing [30] a cough audio classifier with rural inputs for cultural relevance; and Shinyanga’s clear, low-noise environment for optimal cough sound recording.

### Study Implementation

#### Overview

**Figure 1:**
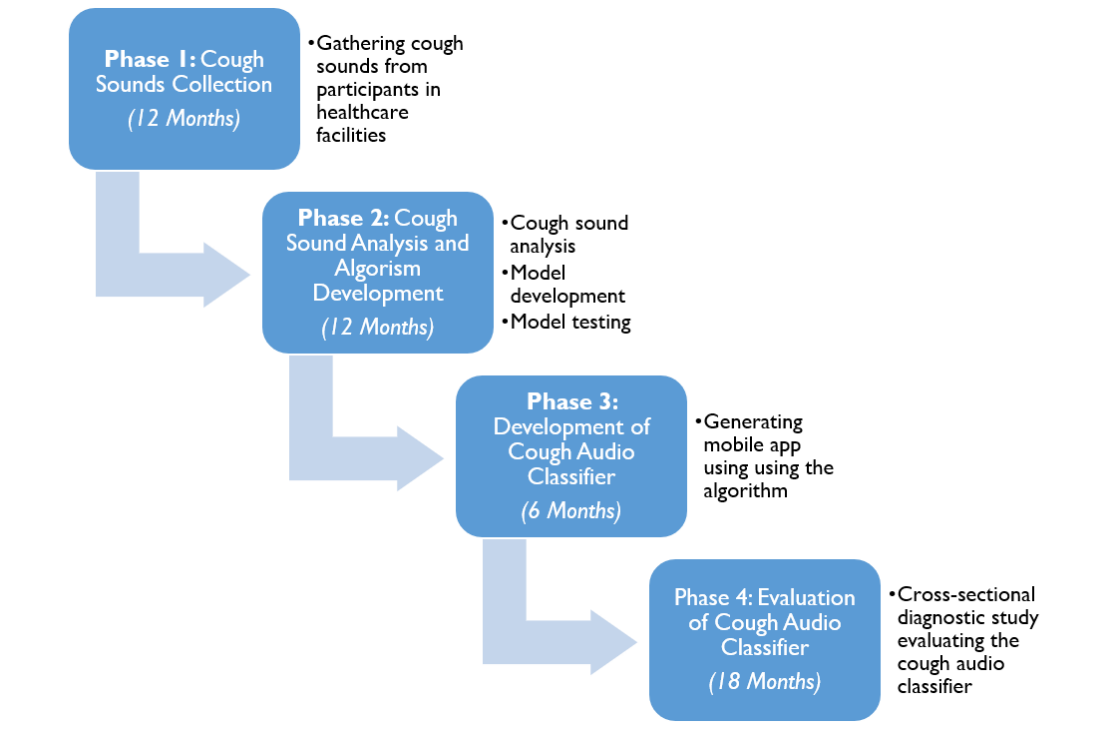
Study implementation framework.

The study implementation is concisely illustrated in Figure 1. A concise summary is provided.

#### Phase 1: Cough Sounds Collection (12 Months)

The procedures for cough sound collection are adapted from previous research in South Africa [12] considering local contexts. More specifically, cough sound data will be collected from health care facilities using specialized cough sound recording devices equipped with high-quality microphones in a dedicated cross-ventilated room. These devices will be nonintrusive and user-friendly, ensuring patient comfort and compliance. Patients with active TB, asthma, and COPD will be identified in the outpatient department (OPD) clinics and directed to the recording room. During the recording sessions, patients will be instructed to cough into the device several times, producing a diverse set of cough sounds. All audio recordings will be conducted by a health care provider, in selected health care facilities during regular clinic hours, who are trained to operate the recording device. The recording room will not be fitted with additional soundproof materials to mimic the clinical environment where the diagnostic procedures for patients with TB, asthma, and COPD are performed. A comprehensive summary of the audio sound capturing process is detailed in Multimedia Appendix 1 [11,12,31-36].

The research team will adhere to ethical guidelines and ensure the privacy and well-being of participants. The inclusion criteria for patients whose cough sounds will be collected include patients who are older than 18 years of age and with a clinical diagnosis of TB, asthma, or COPD diseases. The exclusion criteria will be patients who provide no consent and those unable to provide a cough sound. The decision to exclude children younger than 18 years of age from our study was driven by the extensive consent process required for minors, compounded by our limited resources. Additionally, previous research has highlighted challenges in collecting cough sounds from this demographic [37-39].

#### Phase 2: Cough Sound Analysis and Algorithm Development (12 Months)

Once the cough sound data are collected, advanced signal processing and ML techniques will be used to analyze and classify the cough sounds associated with each respiratory disease. By comparing the acoustic features and patterns among the 3 conditions (TB, asthma, and COPD), the study aims to develop a robust algorithm for automated disease discrimination. A proposed approach for audio classification is using TensorFlow applying Librosa software (Librosa Organization) described previously [11,40]. Librosa is an open-source Python package for music and audio analysis and can provide the data and the sampling rate which plays a vital role in audio classification since different sounds have different sample rates [40]. A comprehensive summary of the procedures for cough sound analysis, algorithm or model development, and testing has been summarized in Multimedia Appendix 2 [11,12,34,35,40-46].

The emerging model will be checked whether it correctly predicts the cough sounds. The expected outcome of this process is a reliable model that can assist the research team in developing a mobile app to help health care practitioners identify TB, asthma, and COPD based on cough sound patterns.

#### Phase 3: Development of Cough Audio Classifier (6 Months)

The developed models for TB, asthma, and COPD will need to be integrated into a noninvasive and user-friendly diagnostic tool. Under the guidance of developers, the development of a mobile app for TB, asthma, and COPD detection will involve the deployment of the developed models to Android with TensorFlow Lite [47]. The sample app will be cloned from GitHub, the sound classification Android app will be imported into Android Studio, and the model will be added (both the soundclassifier.tflite and labels.txt) into the src/main/assets folder replacing the example model that is already there and the app will be built and deployed on the Android device.

#### Phase 4: Evaluation of Cough Audio Classifier (18 Months)

This will be a cross-sectional diagnostic study evaluating the cough audio classifier for active TB, asthma, and COPD triage and case detection against the reference standards. Clients attending OPD clinics at rural health care facilities and suspected of having TB, asthma, and COPD as per national guidelines will be included in the study, upon written consent. Data will be collected from each of the participating facilities. The results of recorded cough using a cough audio classifier will be compared with the current standard of clinical assessment, spirometry, peak expiratory flow (PEF), GeneXpert, culture and sensitivity, chest x-rays (CXR), clinical assessment, and the specificity and predictive values will be calculated.

### Study Design

The study will be a multicenter hospital-based validation cross-sectional diagnostic study evaluating the cough audio classifier for active TB, asthma, and COPD triage and case detection in routine care against the reference standards. This study will involve consecutively enrolled suspects who will be closely evaluated to define their illness. The diagnostic accuracy of automated smartphone-based cough audio will be compared among TB, asthma, and COPD cases and controls. Cases include participants who meet “Definite” or “Probable” TB, asthma, and COPD criteria. Controls include participants who presented with symptoms potentially suggestive of TB, asthma, and COPD, but were not started on treatment and had full clinical resolution at follow-up or had an alternative diagnosis confirmed.

### Study Area

The evaluation study will be conducted in similar study settings and the 30 health care facilities in which cough sounds were collected in Shinyanga Region (phase 1)*.*

### Participants Recruitment

Patients attending OPD clinics and meeting all eligibility criteria listed in the eligibility criteria will be referred to the study’s research staff primarily by the treating medical officer or nurses during triage at OPD. Once enrolled, participants will be retained in the study by telephone reminders. Research assistants will call the participant to arrange follow-up visits and reminder calls will be placed prior to the expected follow-up visit appointments. If there is significant difficulty with participant retention, the possibility of using community health care workers to conduct home visits will be explored. The participant will be reimbursed for the cost of travel to or from the follow-up appointments.

### Eligibility Criteria

#### Overview

There will be no exceptions to eligibility requirements at the time of enrollment. Questions about eligibility criteria will be administered prior to attempting to enroll the participant. The eligibility criteria for this study have been carefully considered. Patients not meeting the criteria will not be enrolled in the study. Participants will be considered eligible for enrollment in this study if they fulfill all the inclusion criteria and none of the exclusion criteria.

#### Inclusion Criteria

The criteria for inclusion are (1) patients aged older than 18 years and presenting with cough; (2) clients attending outpatient clinics in the selected study sites; and (3) clients suspected of having tuberculosis, asthma, and COPD as per national guidelines—prioritizing those with cough of any duration.

#### Exclusion Criteria

The exclusion criteria are (1) refusal to participate and (2) clients who started prescription medications.

### Sample Size Estimation

This evaluative study will collect and analyze data for all patients receiving TB, asthma, and COPD care, starting from screening, and ending with the starting of treatment or preventive therapy. A sample size of 348 for each disease condition (a total of 1044) was obtained using the Buderer formula for sensitivity and specificity [48]. Using tuberculosis as an example, the reported sensitivity of 0.98and specificity of 0.84 of using AI in detecting *Mycobacterium tuberculosis* [49,50] and the prevalence of pulmonary TB of 0.335 among patients with presumptive TB [51] were used to compute this sample size at 0.05 precision and 0.1 attrition.




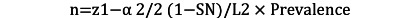




Where n is the required sample size, SN is the anticipated sensitivity, α is the size of the critical region (1−α is the confidence level), Z1−α2⁄ is the standardized normal variate corresponding to the specified size of the critical region (α), and L is the absolute precision.

### Participants’ Service Flow

Patients’ TB, asthma, and COPD service flow all project activities will be consistent with the service standards as outlined in the national policies and guidelines. Currently, in Tanzania, patients’ access to TB, asthma, and COPD services is mainly through facility-based health services. The flow of patients starts with screening for symptoms and signs. Participants who screen positive for TB, asthma, and COPD symptoms (presumptive clients) are either assessed clinically first and referred directly to the laboratory within the same or outside facility for diagnosis (for TB) or receive medical treatment after medical assessments and investigations (for asthma and COPD). Specifically for TB, health providers then order sputum for smear microscopy or GeneXpert and the sample is collected at the referring entry point or at the laboratory, depending on the client volume and human resources at the site. When the sputum sample is negative, a CXR is ordered to support clinical radiological diagnosis. Sputum samples with positive results are sent to the TB clinic service provider who then liaises with the requesting clinician (at various service delivery points) to ensure that the patient receives their result and initiates TB treatment. Patients may be waiting for their result (same-day diagnosis) or be given an appointment to return to the requesting clinician for follow-up. CXR results are interpreted by trained clinicians if available at the facility level, or by the district TB coordinator. For asthma and COPD, a clinical diagnosis may be made by an attending clinician and medications initiated, occasionally after excluding TB where diagnostic facilities are available.

### Clinical Evaluations

A variety of clinical assessments will be conducted, encompassing the collection of demographic and medical data, anthropometric measurements, chest auscultations, spirometry, PEF, and clinical specimen collection. A comprehensive overview of these clinical evaluations is described in Multimedia Appendix 3 [52,53].

### Data Management and Analysis

#### Data Management

Study personnel will be given in-service training by the PI about forms and study procedures before the start of the study and given in-service training periodically throughout the study. Data collection is the responsibility of the research assistants at the site under the supervision of the PI. During the study, the PI will maintain complete and accurate documentation for the study. Data analysis will be the responsibility of the PI, under the mentorship of a biostatistician.

Each subject will be assigned a unique patient ID number. The forms that contain the subjects’ names, dates of birth, and contact information will be kept in the PI’s office and filed in a secured cabinet. A key linking each participant to their patient ID will be created and kept secured by the PI. All case report forms (CRFs) will be reviewed for accuracy, clarity, and completion, and by the data entry staff for completion. Study-related laboratory reports will be reviewed and signed off by a PI. Data reported on the CRF that are derived from source documents or chart reviews should be consistent with the source documents or the discrepancies should be explained.

#### Data Capture

Data will be captured using paper CRFs or electronic CRF and transcribed into a secure, password-protected, electronic database. Data capture will be ongoing throughout the period of the study. It is expected that participant CRFs will be reviewed and entered electronically directly or within 7 days of completion of the visit. Data quality control measures will be instituted as outlined in the standard operating procedures, in accordance with data quality control procedures at the study site.

#### Types of Data

Identifiable information including name and contact information will not be stored electronically, but only on paper. A coded CRF-compliant, secure, password-protected electronic database will house the demographic data, clinical data (including current and past medical history), and laboratory outcome measures (including results of study-related tests or procedures).

#### Analysis for Outcomes

Results of recorded cough using cough audio classifier will be compared with clinical assessment, GeneXpert, culture and sensitivity, spirometry, PEF and sensitivity, and specificity and predictive values will be calculated. These measures will be collected simultaneously among patients and will serve as benchmarks for comparison purposes. The analysis will be generated for cost, facilitators, and barriers to implementing the cough audio classifier for TB, asthma, and COPD screening in rural community settings.

#### Audio Classifier-Diagnostic Test

A binomial test will be used to test whether the sensitivity of the audio classifier using the previously reported cut-off is less than 0.8, and receiver operating characteristic analysis will be explored to determine if another cut-off should be used in this patient population. Logistic regression models will be used to determine whether an audio classifier contributes additional information to predicting TB, asthma, and COPD in the presence of clinical and laboratory predictors. Variable assessment will be based on a likelihood ratio test, in which the likelihood of the reduced model will be compared to the likelihood of the full model. As a primary summary measure of discrimination, we will use the c (for concordance) index, a measure that is identical to the area under the receiver operating characteristic curve when the end point is binary.

#### Risk Factor Analysis

The study will use a similar strategy as described. All models will be adjusted for baseline medical and demographic characteristics of the subjects. Data will be also summarized with respect to the primary and secondary outcome measures, demographics, and baseline characteristics. Quantitative variables will be summarized with standard descriptive statistics while frequency tables will summarize categorical variables. All analyses will be performed in STATA (StataCorp). Before analysis, the outcome will be examined for skew and kurtosis to determine the need for standard transformations to meet normal distribution assumptions. All *P* values are 2-sided and considered statistically significant at a level of .05.

A deductive thematic analysis approach will be used for qualitative data [54] on the barriers and facilitators of cough audio classifier acceptance using NVivo software (Lumivero). More specifically, data transcription and translation will occur simultaneously by the research assistants. After transcription and translation, the interview transcripts will be cross-checked by the PI to ensure that the participants’ worldview was not lost during translation. The interview transcripts will then be deidentified, and pseudonyms generated for each participant. The data will then be uploaded into NVivo software for thematic coding. The PI will deductively generate an initial list of codes from data extracts of the first 3 transcripts. Then, these codes will be reviewed by the research team who had independently reviewed selected transcripts generating a consensual list of codes. The PI will continue coding the rest of the transcripts, refining, and generating more codes upon coming across a new segment of data that could not fit into the initial codes. Codes will then be sorted into potential subthemes and themes, followed by collation of all relevant coded data extracts within identified themes. Throughout coding and refinement, the peer consultation will be maintained to reflect on the subthemes and themes generated.

### Ethical Considerations

The study complies with the principles of the Declaration of Helsinki, as amended by 59th World Medical Association General Assembly, Seoul, Korea, October 2008. It will also be conducted in compliance with the approved protocol, the principles of Good Clinical Practice and applicable local laws and guidelines. The study protocol, information, and consent documents will be submitted to the National Institute for Medical Research (Tanzania) for review and approval. Permission will be sought from the President’s Office Regional and Local Administrative Government (PORALG), the national TB and leprosy programme (NTLP), and participating health facilities. A comprehensive overview of the informed consent process, along with measures for ensuring confidentiality and data protection, is outlined in Multimedia Appendix 4.

## Results

### Participants Demographics

The first section of the results will summarize the demographics of participants involved in cough sound collection and evaluation of the cough audio classifier. Participants’ gender; age; primary occupations; marital status; presence and duration of cough and other TB, asthma, and COPD symptoms and TB contact and family history; current or chronic medications; and comorbidities are some of the demography that will be presented in a tabular form.

### Phase 1: Cough Sounds Collection

The findings will encapsulate a compilation of various cough sounds gathered during the collection process. This compilation will encompass a spectrum of cough characteristics, including frequency, duration, and intensity. Additionally, a summary of the extensive database comprising cough audio recordings, intended for use in subsequent phases, will be provided.

### Phase 2: Cough Sound Analysis and Algorithm Development

The findings regarding the methodology and procedures used to analyze patterns and correlations within the cough sound data set, facilitating algorithm development, will be outlined. Additionally, the process, methodologies, and findings related to the initial algorithm development, distinguishing between different cough sounds, and identifying potential classification markers, will be detailed. Furthermore, a summary of the steps taken to refine the algorithms and improve accuracy and efficiency in identifying distinct cough patterns will be provided.

### Phase 3: Development of Cough Audio Classifier

The findings will encapsulate an overview of the process and methodologies leading to the successful creation of a functional cough audio classifier, designed as a smartphone-based app, derived from the refined algorithms developed in phase 2. The summary will outline the procedures used to assess the classifier’s capacity to accurately differentiate between various types of coughs, specifically identifying those associated with conditions. Similarly, it will highlight the process for evaluating the classifier’s adaptability across a broad spectrum of cough variations and its potential for real-time implementation.

### Phase 4: Evaluation of Cough Audio Classifier

The study will present findings from a performance evaluation aimed at assessing the accuracy, sensitivity, and specificity of the cough audio classifier in a real-world setting. These results will encompass an evaluation of the classifier’s feasibility, acceptability, user experiences, and efficiency among health care providers and patients. Additionally, the study will present findings from a comparative analysis of the cost-effectiveness and practicality of implementing the cough audio classifier in contrast to existing standard practices.

Collectively, these key results from each phase will significantly contribute to a comprehensive understanding of the cough audio classifier’s feasibility, accuracy, and its potential for real-world applicability, as developed within this study.

## Discussion

### Principal Findings

The study aims to enhance the management of TB, asthma, and COPD in health care facilities involved in the research. It aligns with existing patient care pathways for TB, asthma, and COPD by focusing on improving diagnostic capabilities through the integration of services and decentralization, thereby reinforcing referral systems within and between health care facilities. The anticipated primary outcome is the development and validation of a noninvasive, mobile-based cough sound classifier for detecting TB, asthma, and COPD. This research will report on the practical evaluation of the acceptability and effectiveness of the cough audio classifier, with a particular focus on its applicability in rural primary health care settings.

### Comparison to Prior Work

The scholarly community is increasingly interested in the potential of AI or ML technologies for developing diagnostic tools. This research aims to develop and explore the feasibility, acceptability, and efficacy of using AI or ML to create cough audio classifiers in Tanzania, marking the first pilot project of its kind in the region. The results of this study will be benchmarked against findings from previous research on cough audio classifiers, some of which have been reviewed in the context of this protocol [13-22], to provide a comparative analysis and contribute to the evolving field of AI or ML applications in health care diagnostics.

### Strengths and Limitations

This study represents a groundbreaking effort in the development and evaluation of a noninvasive, user-friendly AI-powered cough audio classifier for 3 respiratory diseases within low-income rural settings. However, conducting a study in rural Tanzania may encounter several limitations. First, access to technology poses a significant challenge. Rural areas may lack the necessary technology infrastructure, including stable internet connections, cough audio processors, reliable power supply for devices, or access to smartphones or suitable devices required for the AI-powered application. To mitigate these challenges, efforts will be made to leverage financial and nonfinancial resources within and beyond Aga Khan University. Second, data quality and quantity could be limited. The availability and quality of cough audio data for training the AI model might be insufficient or biased in rural settings, potentially affecting the model’s accuracy. To address this, institutionalized quality assurance measures will be implemented to ensure high-quality data collection. Additionally, the study duration will be adjusted to ensure the collection of sufficient data. Third, language and cultural considerations might impact the classifier’s accuracy. The AI model may not be optimized to recognize local dialects or variations in cough sounds due to cultural or linguistic differences. To partly address this, global AI and ML experts and processing protocols will be used to ensure accurate cough audio processing. Fourth, the study targets adults aged 18 years and older, primarily due to the complexities of the consent process, resource constraints, and the difficulties associated with collecting cough audio sounds from children. Given the pilot nature of this research, subsequent studies may consider including children as participants. Consequently, the study aims to exclude participants who have taken prescription medications. This exclusion criterion is based on the premise that prescription medications can markedly modify the characteristics and severity of cough sounds, thereby influencing the precision of our cough audio classifier. Given that certain prescription medications might be available over-the-counter within the study environments, there is a potential for this criterion to substantially diminish the sample size. In response to this challenge, we have expanded the number of health care facilities involved and extended the period of data collection to mitigate the impact on our sample size and ensure robust data acquisition. Finally, limited health care infrastructure in rural areas, including a scarcity of health care facilities and skilled personnel, may hinder the effective implementation of the AI-powered classifier. To counter this, health care workers involved in the study will receive adequate training before engaging in the research. Recognition is made that addressing these limitations are crucial to ensure the successful development, implementation, and evaluation of the AI-powered cough audio classifier for respiratory condition detection in rural Tanzania.

### Future Directions

The findings of this study will have a range of social impacts. Although there is a lack of common definitions, conceptualizations and practical tools addressing the acceptability of technological health innovations in sub-Saharan Africa [55], an acknowledgment is made that social and cultural contexts have a significant impact on how humans behave both positively and negatively [56]. This is also the case when we are addressing the adaptation to new innovations among health care providers and community members. Common barriers to acceptance of health innovations may include intrapersonal and interpersonal, community, organizational, and policies or enabling factors [57,58]. Intrapersonal factors encompass attitude, fears, level of income, self-efficacy, and education level, which could play both roles as facilitators and barriers when it comes to the acceptability of these new techniques. For example, a recent review noted fear of a loss of professional autonomy and difficulties in integrating AI into clinical workflows as factors hindering their acceptability [57]. Public fears toward health innovations that they are not fully aware of may also promote negativity toward them [58]. Some of the interpersonal factors include social or family roles and responsibilities, norms, stigma, and community influence. Individuals and communities are seen to be more open to innovations when the intervention is supported by influential people within their communities [58]. Challenges are expected on the issue of acceptability among health care workers and the community since it is a new technology introduced and this can cause some reluctance. Additionally, the issue of smartphone use can be a challenge as some potential health care professionals may not be willing to use their personal devices for clinical diagnostic tasks and patients might not own the smartphone where the app will be installed. Similarly, the sharing of smartphones in the family and at the community level for self-diagnosis can be a potential for spreading communicable respiratory diseases if proper hygiene is not observed. Therefore, this study will navigate through these factors and ensure that they are well-addressed to maximize our outcomes. The focus will be on integrating the end users in the early stages of AI development, offering needs-adjusted training for the use of AI in health care and providing adequate infrastructure [57]. Furthermore, health care providers will be trained as trainer of trainers from the start of the study as they tend to easily pave the way, and consideration of gender roles, ensuring both males and females can participate equally.

Furthermore, the findings will have implications on health economics. Scaling up TB, asthma, and COPD services to achieve national and global targets requires an optimization of available funding. To identify and address the available inefficiencies and consider new interventions at the national level; information on the cost of services is of great necessity. There is a scarcity of data on the cost and technical efficiency of using mobile health (mHealth) for early TB, asthma, and COPD detection in rural communities that once done can aid in the achievement of national goals. Thus, we aim to gather health economics data to assess total and unit costs, cost structure, and cost drivers of this technology provision. The purpose of the health economics component of this study is to inform priority setting within the health sector. This will cover resource allocation across an overall budget or total resource envelope from the health provider’s perspective that focuses on costs incurred by the implementing partner but also assesses the costs of services and consumables from other providers (eg, national program) in both financial and costs.

Patient and household expenditures including productivity losses have been barriers to accessing and retention in care. In consultations with health economists, a plan is to collect data on both direct and indirect patient-incurred costs using questionnaires. The study is expected to estimate direct costs incurred by patients such as those related to transport, as well as indirect costs, in terms of productivity losses, related to access to care as well as due to illness-related absenteeism. Together with data on service usage and socioeconomic background, we will be able to analyze patient costs and equity considerations in access and use of care. The study will generate a comprehensive picture of the costs of using mHealth-based cough audio triage testing for active pulmonary TB, asthma, and COPD in health facilities in high-burden settings in Tanzanian communities.

### Dissemination Plan

The dissemination of findings will involve communication with key stakeholders, regular reporting through the pertinent channels of the funding agency and the Tanzanian Ministry of Health, engagement in local and international forums, and publication through peer-reviewed open-access journals. By using a methodical and systematic approach to disseminating the study’s findings, this research aims to contribute to the expanding knowledge base regarding the use of AI and ML in developing noninvasive diagnostic tools. These tools are designed to assist health care professionals in effectively identifying and distinguishing various diseases, not only within Tanzania but also broadening their application beyond geographical boundaries.

### Conclusions

Cough sound classifiers harness the power of advanced technology to offer a promising solution for the early detection and efficient management of various respiratory conditions. By accurately analyzing cough sounds, these innovative tools can identify potential illnesses at an earlier stage than traditional diagnostic methods, facilitating timely intervention and reducing the need for more invasive procedures. This approach not only has the potential to significantly alleviate the burden on public health systems by streamlining the diagnostic process but also aims to improve patient outcomes through faster and more accurate diagnosis. Moreover, the use of cough sound classifiers can democratize health care access, particularly in under-resourced areas, by providing a cost-effective and easily deployable tool for community health workers and primary care settings. Ultimately, the integration of this technology into clinical practice could transform the landscape of respiratory disease management, offering a scalable and efficient solution to global health challenges.

## Appendix

Multimedia Appendix 1 Procedures for capturing cough sounds.

Multimedia Appendix 2 Procedures for model development.

Multimedia Appendix 3 Guidelines for clinical evaluations.

Multimedia Appendix 4 Informed consent process, confidentiality, and data protection.

Multimedia Appendix 5 Sample transcript of conversation with generative artificial intelligence.

## Abbreviations

AI: artificial intelligence
COPD: chronic obstructive pulmonary disease
CRF: case report form
CXR: chest x-ray
HC: health centre
mHealth: mobile health
ML: machine learning
NTLP: national TB and leprosy programme
OPD: outpatient department
PEF: peak expiratory flow
PI: principal investigator
PORALG: President’s Office Regional and Local Administrative Government
TB: tuberculosis
WHO: World Health Organization

## Data Availability

Some data sets generated and analyzed during this study will not be publicly available due ethical reasons and a need to protect participant privacy but will be available from the corresponding author on reasonable request.
