# Supplementary material for: Leveraging AI and Machine Learning to Develop and Evaluate a Contextualized User-Friendly Cough Audio Classifier for Detecting Respiratory Diseases: Protocol for a Diagnostic Study in Rural Tanzania
Source: JMIR Res Protoc. 2024 Apr 23;13:e54388. doi: 10.2196/54388 (PMC11077412; doi:10.2196/54388)
Supplement: Multimedia Appendix 1 [file resprot_v13i1e54388_app1.docx]

**Study title:** **Leveraging Artificial Intelligence and Machine Learning to Develop and Evaluate a Contextualised User-Friendly Cough Audio Classifier for Detecting Respiratory Diseases: A Protocol for a Diagnostic Study in Rural Tanzania.**

**APPENDIX 1: PROCEDURES FOR CAPTURING COUGH SOUNDS**

Within cross-ventilated rooms (Figure 1), the audio cough sound will be captured using a sensitive automatic noncontact cough detector (Zoom F8n Filed recorder) connected to a computer and simultaneously recorded in real-time using a digital SONY audio tape recorder which will serve as serves as a backup. Both microphones will be covered with standard N95 face masks that will be discarded after each patient. Covering a cough audio recorder with a face mask has been documented to not affect the quality of the recorded signal substantially [12]. Likewise, the use of non-contact microphones is preferred because contact microphones have been documented to suffer from reliability issues [31-32]. The study healthcare professional will ensure that a patient maintains a gap of at least 10 to 15 centimeters between the patient and the microphone. Each patient will be asked to count from one to ten, cough, take a few deep breaths and then cough again, thus producing at least four bursts of cough. The choice of four coughs was based on experiences from previous studies [12,33-34]. Since the patients in this study will have active diseases, prompting them to cough is expected to elicit irritation of the respiratory system producing a cough sound which mimics a voluntary cough in a non-research scenario.


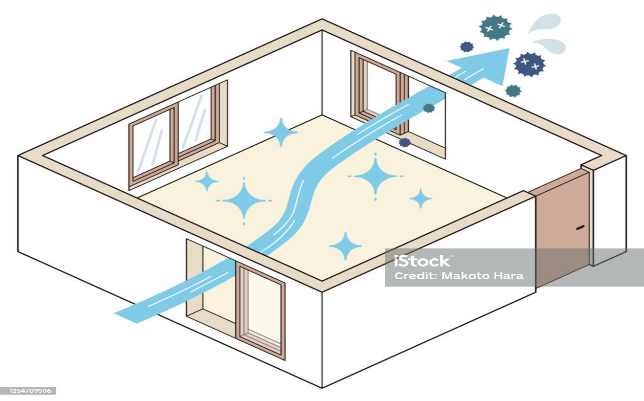

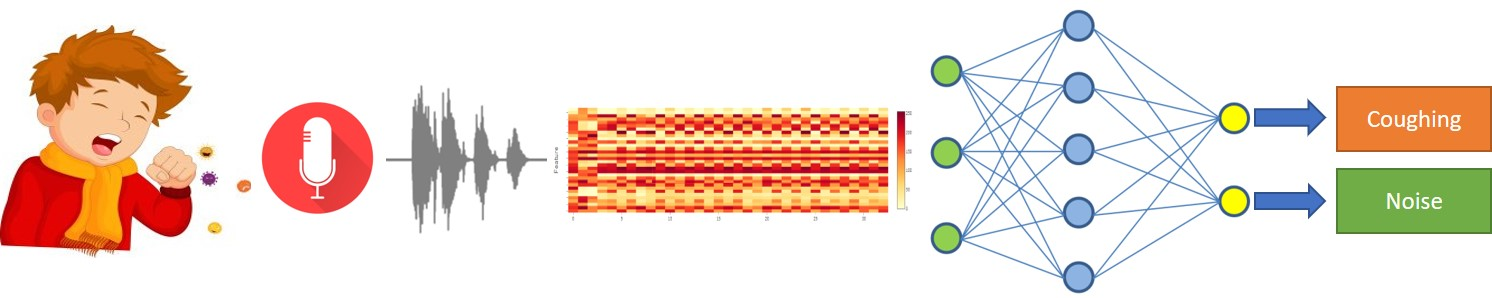


Figure 1: Cross-ventilated room (Image source: iStock.com). Figure 2: Cough Sound Capturing (Image source: sensiml.com)

All audio recordings will be sampled at between 16kHz which falls within audible range for humans [11]. The portions of the resulting audio recordings that contain coughing will be manually annotated using the EUDICO Linguistic Annotator (ELAN) multimedia software [35]. ELAN is computer [software](https://en.wikipedia.org/wiki/Software) used by professionals to manually and semi-automatically annotate and [transcribe](https://en.wikipedia.org/wiki/Transcription_(software)) audio or video recordings [36]. Additionally, relevant demographic, and clinical information, such as age, gender, medical history, disease severity and investigations made, will be collected.
