# Supplementary material for: Leveraging AI and Machine Learning to Develop and Evaluate a Contextualized User-Friendly Cough Audio Classifier for Detecting Respiratory Diseases: Protocol for a Diagnostic Study in Rural Tanzania
Source: JMIR Res Protoc. 2024 Apr 23;13:e54388. doi: 10.2196/54388 (PMC11077412; doi:10.2196/54388)
Supplement: Multimedia Appendix 2 [file resprot_v13i1e54388_app2.docx]

**Study title:** **Leveraging Artificial Intelligence and Machine Learning to Develop and Evaluate a Contextualised User-Friendly Cough Audio Classifier for Detecting Respiratory Diseases: A Protocol for a Diagnostic Study in Rural Tanzania.**

**APPENDIX 2: PROCEDURES FOR COUGH SOUND ANALYSIS, ALGORITHM DEVELOPMENT AND MODEL TESTING**

1. Cough Sound Analysis

The process cough sound analysis will commence with creating a new data set and loading the annotated cough audio data into Kaggle; conducting exploratory data analysis (EDA) by installation of Librosa, loading external data on Kaggle into Google Colab and ensuring that the data is good to use. This will be followed by data processing involving feature extraction using the [Mel-Frequency Cepstral Coefficients (MFCC)](http://practicalcryptography.com/miscellaneous/machine-learning/guide-mel-frequency-cepstral-coefficients-mfccs/) algorithm. Introduced by Davis and Mermelstein, the MFCC algorithm has been used very successfully as a feature in audio analysis particularly in automatic speech and speaker recognition since the 1980s [12, 41]. MFCC algorithm summarizes the frequency distribution across the window size enabling the analysis of both the frequency and time characteristics of the provided sound. The MFCC has been found to be useful for differentiating dry coughs from wet coughs [43].

The standard procedures for the computation of MFCCs will include:

1. Extracting patterns/features from audio files using the MFCC algorithm based on the frequency and time characteristics.
2. Creating a list to store all features extracted.
3. Converting the entire extracted feature list into a data frame using the Pandas library which enables conversion of the results into tables. This will result in extracted features and their respective classes for more straightforward analysis [11,40].
4. Splitting the dataset into independent and dependent datasets, x and y;
5. Importing the datasets to_categorical and LabelEncoder methods from TensorFlow and Sklearn.
6. Using sklearn’s train_test_split method to split the dataset into training and test sets.
7. Model building.

The processed cough data will then be used to build a model using TensorFlow. TensorFlow is an end-to-end open-source platform for ML and has a comprehensive, flexible ecosystem of tools, libraries, and community resources that lets researchers push the state-of-the-art in ML and developers easily build and deploy ML-powered applications [44]. TensorFlow core libraries are considered the lower-level libraries that provide APIs to build, train and deploy ML models that can be used for vision and natural language processing (NLP) [44-46]. Building a model will involve:

1. Importing the processed data into the TensorFlow notebook.
2. Training the model considering the number of epochs that are sufficient to guarantee accuracy.
3. Computing validation accuracy. A focus is to obtain a validation accuracy of >80% using values used in previous studies as benchmarks [ 12, 34-35] and the number of training epochs will be increased as needed to achieve a higher accuracy score.

We recognize that the model may achieve an accuracy threshold of 80% for certain diseases while failing to reach this benchmark for others. Accordingly, we will deem the model accurate specifically for those diseases where the target accuracy is attained and acknowledge its limitations for conditions where it falls short. Furthermore, there exists the potential that the model might display reduced validation accuracy uniformly across all three conditions, which could lead to interpretations of its general inadequacy. We are fully prepared to accept and critically analyze any outcomes that arise from the validation process. Our dedication lies in the transparent disclosure of all results, adhering to the principle that findings, irrespective of their nature, contribute valuable insights to the scientific community.

1. Model testing.

To test the algorithm/model, three steps will be performed. First, preprocessing the test audio data by extracting the features using the MFCC algorithm. Second, predicting the test audio class with the help of the model created. Third and final, inversing and transforming the predicted label to get a class label. More specifically, the initial steps (Appendix 2) will be repeated to preprocess audio data followed by performing a prediction of the class it belongs to and finally using the inverse_transform method from scikitlearn to obtain the predicted label name.

It is important to note that the emphasis of our study is centered on validating the efficacy of our audio classifier as an initial screening tool for the identification of diseases. Nonetheless, it is conceivable that findings related to the intricacies of disease staging may surface. Should our model inadvertently exhibit the capacity for precise disease staging, these outcomes would be regarded as a substantial and fortuitous enhancement to our research. Such an unforeseen advancement would surpass our preliminary anticipations and catalyze further avenues for exploration and innovation within the realm of AI/ML-based medical diagnostics.
