# Supplementary material for: Leveraging AI and Machine Learning to Develop and Evaluate a Contextualized User-Friendly Cough Audio Classifier for Detecting Respiratory Diseases: Protocol for a Diagnostic Study in Rural Tanzania
Source: JMIR Res Protoc. 2024 Apr 23;13:e54388. doi: 10.2196/54388 (PMC11077412; doi:10.2196/54388)
Supplement: Multimedia Appendix 3 [file resprot_v13i1e54388_app3.docx]

**Study title:** **Leveraging Artificial Intelligence and Machine Learning to Develop and Evaluate a Contextualised User-Friendly Cough Audio Classifier for Detecting Respiratory Diseases: A Protocol for a Diagnostic Study in Rural Tanzania.**

**APPENDIX 3: GUIDELINES FOR CLINICAL EVALUATIONS**

The following clinical evaluations will be performed, with further detail provided in the standard operating procedures (SOP). A comprehensive overview is as follows:

1. Interview for demographic and medical information.

Once full permissions have been obtained, the participant will undergo a medical interview and assessment, and specific study procedures. The medical interview and assessment is expected to take less than 30 minutes. Specimen collections are expected to take less than 30 hours. X-ray services will be utilised where available and when clinically recommended to minimise the cost of travel to X-ray facilities. Participants will be asked to have at least 3 visits (at time=0, 1 and 2 weeks) for interviews, anthropometrics, and specimen collections. Demographic and clinical information will be gathered verbally at baseline via participant interview by the Research Assistant and recorded on the “Medical Interview Form” (MIF): age, gender, presence and duration of cough and other TB, Asthma and COPD symptoms and TB contact & family history, current/chronic medications, and co-morbidities. The study doctor will review this information as per the study guidelines. Similar information will be gathered at the follow-up visits via interview of the participant with a specific emphasis on symptom resolution and medication management/initiation.

1. Anthropometrics:

All participants will have anthropometric measurements obtained at baseline and subsequent visits. The Research Nurse will measure the subject’s height to the nearest 0.1cm using a locally available height stick. Similarly, weights to the nearest 0.1kg will be obtained while wearing light clothes using a locally available electronic scale. Measurements will be recorded on the Clinical Review Form (CRF).

1. Chest Auscultations

The study doctor will conduct auscultations to determine the breath sounds. The findings will be recorded in the procedure form (above)

1. Spirometry and Peak expiratory flow (PEF)

Spirometry and PEF tests that are recommended for Asthma and COPD diagnosis [52-53] will be performed and documented.

1. Chest X-Ray (CXR)

Where available, CXR will be conducted for all patients enrolled on the study. The project will meet the cost of CXR for patients. The project may not be able to meet the cost of CXR to a different facility for all patients due to the geographical limitations of the study settings.

1. Specimen Collection and investigations

The study will utilise existing healthcare facility personnel and laboratory infrastructure for sample collection and testing (AFB smear, GeneXpert and culture) adhering to the national SOPs. In facilities where sample testing is not possible, the laboratory personnel will be involved in sample collection and transportation to the reference laboratory ( the Shinyanga Regional Referral Hospital) and reimbursed associated costs.
