# Supplementary material for: Leveraging AI and Machine Learning to Develop and Evaluate a Contextualized User-Friendly Cough Audio Classifier for Detecting Respiratory Diseases: Protocol for a Diagnostic Study in Rural Tanzania
Source: JMIR Res Protoc. 2024 Apr 23;13:e54388. doi: 10.2196/54388 (PMC11077412; doi:10.2196/54388)
Supplement: Multimedia Appendix 4 [file resprot_v13i1e54388_app4.docx]

1. Informed Consent Process

The investigators will choose subjects in accordance with the eligibility criteria detailed above. All subjects will sign an informed consent form that complies with the requirements of CFR. An extensive discussion of the risks and possible benefits of this therapy will be provided to the subjects. They will receive a comprehensive explanation of the proposed procedures, including the nature and risks of the study. Sufficient time will be allowed to consider participation in the study after having the nature and risks of the study explained to them and having the opportunity to discuss the study with their family, friends or legally authorized representative or think about it prior to agreeing to participate. Consent forms describing in detail (in Swahili) the study procedures, and risks will be given to the subject. Consent forms will be IRB-approved, and the subject will be asked to read and review the document. Upon reviewing the document, the investigator or designee will explain the research study to the subject and answer any questions that may arise. Subjects must sign the informed consent form and written documentation of the informed consent process is required prior to starting any study procedures.

Potential study participants will be referred to the study’s research staff primarily by the treating medical officer. Study staff will regularly liaise with these clinical staff members to ensure complete capture of patients newly suspected of having TB, Asthma and COPD. Medical officers will provide the subject’s name to the research staff. Subjects will be approached by the research staff fluent in Kiswahili and English and invited to assess interest in study participation. This discussion will take place in the privacy of a dedicated clinic room. If a subject expresses interest in potentially participating, they will be given a full description of the study. The final approved English language consent documents will be translated into Swahili with appropriate back-translations for accuracy. The study, including the reason for the study, full study process (including the need for three visits), and all study procedures (which will include cough sounds, brief interviews, anthropometrics, spirometry, PEF, socio-science and specimen collection at baseline, risks, benefits, alternatives, and patient rights, will be described. The subject’s level of understanding will be assessed verbally during and at the conclusion of the consent process.

In the event that a subject is illiterate in Kiswahili, the consent form will be read aloud and if the subject agrees, a thumbprint will be requested in place of a signature with a witness present. A copy will be given to the subject for their records. All subjects will have the right to withdraw their consent at any time throughout the course of the study.

By signing the informed consent form, the subject agrees to complete all evaluations required by the study, unless the subject withdraws voluntarily or is terminated from the study for any reason. The rights and welfare of the subjects will be protected by emphasizing to them that the quality of the local medical care will not be adversely affected if they decline to participate in this study.

1. Subject Confidentiality

The study will be conducted in compliance with the approved protocol, and the World Medical Association Declaration of Helsinki entitled “Ethical Principles for Medical Research Involving Human Subjects” (2013 version) and in compliance with all local ethical, regulatory, and legal requirements in Tanzania. Consent forms will be stored under the supervision of the site PI in a secured office and accessible to study staff only. Research samples are collected and managed by the healthcare facility laboratory staff for laboratory diagnostic evaluation. The utmost effort will be made to avoid a breach of patient confidentiality, in all discussions with study staff, handling of documentation, and management of the study data, as far as possible within the law. This confidentiality is extended to the results of laboratory tests, with the provision that since TB is a notifiable disease in Tanzania, it is the investigators’ legal obligation to refer participants with TB to the local health services. This requirement is stipulated in the information and consent document. No other information concerning study participants will be released to any third party, for example, the participant’s general practitioner, without prior written approval from the participant. If the participant gives their written consent to do so, participant contact details may be used by the PI who might wish to contact the participant to take part in future research.

1. Protection of electronically stored participant data

Participants will be allocated a participant identification number (PID) which will be used to protect their identity. The secure (password protected) REDCapTM database will only be accessed by members of the research team. The REDCapTM database will be coded with the PID only. A separate log will be kept securely at each site linking the PID to patient identifiable information (name, date of birth); this will take the form of a password-protected, Microsoft Excel spreadsheet saved on a secure server at each site. This system (sometimes called ‘pseudonymised’ data) ensures that confidentiality is protected during routine collection of data. Only the pseudonymised database will be used during the analysis. The electronic database will be kept securely in pseudonymised form for 7 years following the end of the study in accordance with relevant legislation.

Paper documents, such as clinical notes, laboratory data and administrative documentation will be kept in a secure location, with access restricted to people involved in the research (for example, locked filing cabinets in a room with restricted access) and held for 7 years after the end of the study. During this period, all data will be accessible to the competent or equivalent authorities, the Sponsor, (and other relevant parties) with suitable notice. The data may be subject to an audit by the competent authorities.
