# Supplementary material for: Leveraging AI and Machine Learning to Develop and Evaluate a Contextualized User-Friendly Cough Audio Classifier for Detecting Respiratory Diseases: Protocol for a Diagnostic Study in Rural Tanzania
Source: JMIR Res Protoc. 2024 Apr 23;13:e54388. doi: 10.2196/54388 (PMC11077412; doi:10.2196/54388)
Supplement: Multimedia Appendix 5 [file resprot_v13i1e54388_app5.pdf]

ChatGPT 3.5

specify a date for the commencement of data collection at this time.

KA

You

Refine: We confirm that authors information is correct

ChatGPT

We confirm that the authors' information is accurate.

KA

You

Refine:

While we comment the editorial work done, the references need to be fixed to ensure correctness. Considering the initial approach of having separate list for multimedia appendices may minimize the potential miscitation.

ChatGPT

We appreciate the editorial efforts made; however, the references require adjustment to ensure accuracy. We suggest maintaining separate lists for the multimedia appendices to minimize the potential for miscitation.

Message ChatGPT...

ChatGPT can make mistakes. Consider checking important information.
